# Astrocytes in Flavivirus Infections

**DOI:** 10.3390/ijms20030691

**Published:** 2019-02-06

**Authors:** Maja Potokar, Jernej Jorgačevski, Robert Zorec

**Affiliations:** 1 Laboratory of Neuroendocrinology—Molecular Cell Physiology, Institute of Pathophysiology, Faculty of Medicine, University of Ljubljana, Zaloška 4, 1000 Ljubljana, Slovenia; jernej.jorgacevski@mf.uni-lj.si; 2 Celica BIOMEDICAL, Tehnološki park 24, 1000 Ljubljana, Slovenia

**Keywords:** astrocytes, flavivirus, neuroinfections, TBEV, ZIKV, WNV, JEV, encephalitis

## Abstract

Virus infections of the central nervous system (CNS) can manifest in various forms of inflammation, including that of the brain (encephalitis) and spinal cord (myelitis), all of which may have long-lasting deleterious consequences. Although the knowledge of how different viruses affect neural cells is increasing, understanding of the mechanisms by which cells respond to neurotropic viruses remains fragmented. Several virus types have the ability to infect neural tissue, and astrocytes, an abundant and heterogeneous neuroglial cell type and a key element providing CNS homeostasis, are one of the first CNS cell types to get infected. Astrocytes are morphologically closely aligned with neuronal synapses, blood vessels, and ventricle cavities, and thereby have the capacity to functionally interact with neurons and endothelial cells. In this review, we focus on the responses of astrocytes to infection by neurotropic flaviviruses, including tick-borne encephalitis virus (TBEV), Zika virus (ZIKV), West Nile virus (WNV), and Japanese encephalitis virus (JEV), which have all been confirmed to infect astrocytes and cause multiple CNS defects. Understanding these mechanisms may help design new strategies to better contain and mitigate virus- and astrocyte-dependent neuroinflammation.

## 1. Introduction

**Figure 1:**
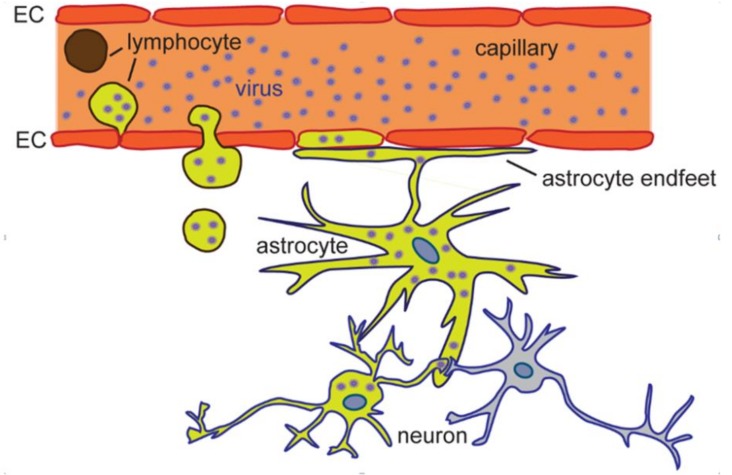
Astrocytes are among the first cells to be infected in the central nervous system (CNS). Viruses (denoted as dots) that circulate within blood vessels can enter the CNS by crossing the blood–brain barrier, which is composed of endothelial cells (EC). The endfeet of astrocytes closely align ECs, thus making astrocytes very receptive to virus uptake. Virus-infected cells are highlighted in olive.

Astrocytes are abundant, heterogeneous neuroglial cells in the central nervous system (CNS) that provide crucial support for the functioning of neurons and hence contribute to brain information processing [1,2]. In general, astrocytes are considered the main cells that provide homeostasis [2] with many key functions, and that act as supporters of neuronal functions by modulating the extracellular levels of neurotransmitters, gliotransmitters, and ions, by regulating synaptogenesis, by providing energy and metabolic support to neurons, by maintaining the blood–brain barrier (BBB) and also by removing waste material via the glymphatic system [2,3,4,5,6,7,8,9,10,11,12,13]. Astrocytes occupy a relatively large part of the brain parenchyma and in addition to enwrapping neuronal synapses, they are in contact with brain capillaries, ventricle cavities, and pia. Close apposition of astrocyte endfeet with the vascular endothelial cells of brain capillaries and pia (Figure 1), appears to make them central in mediating virus infections and infections by other agents [14].

Several viral families, including flaviviruses, have members that are neurotropic, i.e., they have the ability to infect neural tissue [15]. In general, like other neurotropic viruses, flaviruses first trigger viremia (they enter the bloodstream), and when a high level of virus particles is reached in the blood (high viremia), they can invade the CNS [16,17]. Several possible routes are considered to mediate the entry of flaviviruses into the CNS. These include (1) infection of peripheral nerves and olfactory neurons where retrograde transport delivers viruses into the spinal cord and the brain, respectively; (2) traversing the epithelium of endothelial cells of brain capillaries in virus-infected lymphocytes or by direct infection of the endothelial cells by breaching the BBB; (3) breaching the barrier between meningeal blood and cerebrospinal fluid (CSF, blood–CSF barrier), consequently infecting (entering) the CSF; and (4) breaching of the blood–CSF barrier by virus-infected leukocytes or by viruses present within blood vessels of the choroid plexus (CHP) [15,16,17]. Once in the brain, a virus may become neurovirulent, meaning that it is able to replicate and cause damage within the CNS [17]. After entry into the CNS, astrocytes are among the first cell types to come in contact with or intercept the virus; these cells form a structure termed glia limitans, lining the brain capillaries and pia mater (Figure 1). The nature of astroglial involvement in retaining and spreading the virus throughout the brain and astrocyte-mediated pathogenicity of virus-infected CNS is unclear. However, in recent years, studies focusing on how astrocytes respond to flavivirus infections have provided a new perspective on astrocytes in virus infections. While it is known that neurologic symptoms emerge from virus-injured neurons due to neurocytotoxicity of the virus and the induction of an immunopathogenic response [17], the contribution of flavivirus-infected astrocytes to neuropathogenesis has emerged as significant. Recent studies revealed that the infection of astrocytes is important for retention of the virus in the CNS, i.e., for virus production, for spread of the virus to neurons and other cells, and for the immunologic response [18,19] as addressed in the next section.

## 2. Neurotropic Flaviviruses that Infect Astrocytes

Astrocytes can be infected with several flaviviruses, including those that cause severe neurologic symptoms, on an epidemic scale. These viruses can be transmitted to humans from mosquitos (mosquito-borne viruses), ticks (tick-borne viruses), and from yet unknown vectors [17]. Human-to-human transmission has also been confirmed [20]. Genus *Flavivirus* (family *Flaviviridae*) consists of single positive-stranded RNA viruses enclosed within a lipid envelope, carrying attachment molecules (E and M proteins), considered to be required for binding to host cell receptors [17]. Once sequestered into intracellular membrane-bound vesicles, they traffic in the cytoplasm along the endocytotic pathway [19,21,22,23,24,25,26]. The trafficking of endocytotic vesicles in astrocytes can be altered in numerous pathologic conditions [27,28,29]. Once in endosomal compartments, viruses travel along the host cell cytoskeleton until fusion of the virus envelope and endosomal membrane results in the release of the viral nucleocapsid into the host cytoplasm, followed by translation, replication, and production of new virions [17,30]. As described below, for all these processes, viruses appear to hijack several cellular proteins and target organelle membranes. In recent years, the impact of several flaviviruses on astrocyte morphology and physiology has been studied. From the anatomic position of astroglia and their homeostatic role in the CNS, one can predict that virus invasion may lead to important functional consequences for the entire CNS upon interaction of astrocytes with viruses (Figure 1).

Given that neurotropic virus infections pose a significant health hazard and consequential socio-economic burden, neurotropic viruses of interest include tick-borne encephalitis virus (TBEV) (with 10,000–15,000 cases of tick-borne encephalitis every year in Europe and Asia), mosquito-borne viruses including Zika virus (ZIKV; linked to an outbreak of microcephaly with a 20-fold increase in human birth defects in 2016 and estimated to cause more than three quarters of a million suspected and confirmed infections in the Americas), West Nile virus (WNV; with over 15,000 cases of encephalitis annually and millions of people infected) and Japanese encephalitis virus (JEV), which endangers nearly half of the human population with an annual incidence up to 50,000 and one third mortality rate [17,19,31,32,33,34,35,36,37,38].

### 2.1. Tick-borne Encephalitis Virus

TBEV is an important human pathogen that may result in dangerous neuroinfections (meningitis, inflammation of the meninges; meningoencephalitis, inflammation of the brain and meninges; myelitis, inflammation of the spinal cord). It is endemic in Europe and in Asia, mirroring the natural habitat of its principal vectors *Ixodes ricinus* (sheep tick) and *Ixodes persulcatus* (taiga tick) [16,17,33]. Similar to other flaviviruses, the pathways and mechanisms of TBEV invasion of the CNS are largely unclear. Similarly, the neurologic pathogenic potential of TBEV-infected astrocytes is unknown. However, infection of astrocytes with TBEV has been confirmed in recent years, first in primary rat astrocytes, followed soon after by human and mouse astrocytes [18,19,31,32]. TBEV successfully replicates in astrocytes, reaching a higher virus load (TBEV RNA copies/cell) than in cells used for the preparation of virus stocks (i.e., Vero E6 cells) [19,31,32]. The finding that TBEV is successfully replicated in astrocytes was important because several species of small rodents are TBEV-amplifying hosts and have the potential to maintain TBEV through latent persistent infections [17,39]. TBEV was detected in different rodent organs for longer periods after infection, reaching a very high TBEV RNA virus load specifically in the brain [40]. High virus load and long-term presence of the virus in the rodent brain is intriguing. It was reported that primary rat astrocytes show high resilience on TBEV infection; their viability remained unaltered for 14 days after infection [19]. This finding indicates that astrocytes epitomize a potential mediator of brain infection and a reservoir of brain TBEV in rodents. In humans, brain astrocytes may have a similar role.

Interestingly, the viability of primary human brain cortical astrocytes was predominantly unaffected by TBEV infection, although some infected cells underwent necrotic cell death [31]. Despite relatively high resistance of astrocytes to TBEV-triggered cell death, TBEV infection induces several morphologic and physiologic changes in infected rat and human astrocytes. These include changes that enhance virus entry into the host cell (possibly involving changes in the expression of TBEV receptors on host cells), alterations in TBEV-laden vesicles in respect of size and mobility dynamics along the cytoskeleton, changes in actin and tubulin cytoskeleton polymerization, and extensive morphologic changes in the endoplasmic reticulum, Golgi complex, mitochondria, and phagosomes [19,31]. In addition to primary astrocytes, TBEV infection of glioblastoma cells also induced rearrangement of rough endoplasmic reticulum and disintegration of cytoskeletal filaments [18]. The consequences of TBEV-induced changes of the cytoskeleton need to be studied in detail, because cytoskeleton alterations have a significant effect on vesicle traffic in the host cell [41]. Endocytotic TBEV-laden vesicles move along molecular motor-associated filaments; their speed corresponds to the speed of processive myosins along actin filaments and kinesins along microtubules [19]. Interestingly, their mobility increases over longer periods after infection, probably indicating changes in local virus-induced protein synthesis that enhance vesicle trafficking [19,42]. Strategies to modify vesicle traffic in TBEV-infected astrocytes may be used to alleviate TBEV brain insults.

Persistent infection with TBEV induces the formation of extensive membranous tubule-like structures of the rough endoplasmic reticulum; the purpose of these remains to be elucidated [31,43]. It is possible that these protrusions are associated with recently discovered morphologic membrane modifications induced by the adhesion of nanofibers on the bacterial surface [44]. In addition to altering organelle membranes, TBEV triggers astrocyte activation (similar to other flaviviruses), confirmed by increased production of glial fibrillary acidic protein (GFAP), a marker for reactive astrogliosis [45]. Moreover, upon virus infection, astrocytes as well as immune cells and other cell types in the CNS (microglia, oligodendrocytes, myelinating cells of the CNS, endothelial cells of the brain microvasculature, and neurons) release inflammatory cytokines and chemokines [31,46,47,48,49]. These, in combination with substantial virus production in astrocytes, can lead to deterioration in the viability of neurons and may invoke a more severe course of the disease with a higher fatality rate [50,51]. For instance, human brain cortical astrocytes have been shown to significantly increase the expression of pro-inflammatory cytokines and chemokines after TBEV infection; this increase was transient and coincided with the peak increase in TBEV RNA [31].

Clearly, altered TBEV-mediated morphology and physiology of astrocytes may significantly affect the functioning of neighboring cells. In addition to affecting neurons, TBEV infection of astrocytes may also alter the permeability of the BBB, as shown in mice [52]. Because astrocytes tightly line the endothelial cell layer and help maintain the BBB, alteration in astrocyte functioning affects the permeability of the BBB [3,53]. The specifics of this contribution remain to be investigated. Nevertheless, first indications show that disruption of the BBB occurs after TBEV enters the CNS [52] and is largely influenced by cytokine/chemokine overproduction in the brain [52]. Screening of the production of various cytokines/chemokines by astrocytes will elucidate their role in neuroinflammation. So far, measurements of the expression of interleukin 1β (IL-1β), interleukin 6 (IL-6), interleukin 8 (IL-8), interferon α (IFN-α, tumor necrosis factor α (TNF-α), macrophage inflammatory protein 1 β (MIP-1β/CCL4) and interferon γ-induced protein 10 (IP-10/CXCL10) mRNAs revealed upregulation on TBEV infection of human astrocytes [31,53]. One of the key molecules that affects the integrity of the BBB in neuroinflammatory diseases may also be the matrix metalloproteinase 9 (MMP-9), which is significantly overproduced in TBEV-infected astrocytes *in vitro*, and increased in the serum and CSF of TBEV-infected patients [31,54,55].

In addition to contributing to neurotoxicity, neuroinflammation, and disintegration of the BBB, astrocytes can also act protectively [32]. It was recently shown that TBEV infection of mouse astrocytes initiated a rapid interferon (IFN) response through upregulation of type I IFNs, which restricted viral replication and virus spread, all beneficial to survival of astrocytes and neurons in certain regions of the CNS [32,56].

In summary, acute and persistent TBEV infections of astrocytes significantly affect neuronal viability (Table 1). Because astrocytes are an important reservoir of brain TBEV (at least in rodents) and mediators of neuroinflammation, several cellular processes in astrocytes, such as virus entry, traffic of virus-laden vesicles and virus multiplication, are appealing targets for novel therapeutic interventions. 

### 2.2. West Nile Encephalitis Virus

WNV was first discovered in 1937 in Uganda, and it was reported to cause dengue-like illnesses until the end of the 20th century, when its highly neuroinvasive strains initiated large outbreaks with severe cases of WNV encephalitis (WNVE) in Europe and North America [58,59,60,61,62,63]. After an acute phase, patients infected with WNVE can suffer from consequences of the infection for a long time. About 40% of patients recovering from WNVE have long-term physical, cognitive, and functional impairments (muscle weakness, loss of concentration, confusion, and light-headedness) for a year to even a decade after infection [64,65,66]. The nature of such long-lasting pathogenic consequences of WNVE need to be elucidated, as well as patterns of WNV entry into the CNS. One of the recognized routes for entry of WNV into the brain is through the BBB; therefore it is not surprising that astrocytes, being tightly associated with the BBB, are one of the most important cells that are infected with WNV before infection of neurons, as in infection with TBEV [67,68,69,70,71]. Therefore, it is important to understand astrocyte responses to WNV infection in order to understand their influence on neurons in virus-infected brain tissue.

Although it has been confirmed that neurons and glial cells in the human CNS can be infected with WNV, the detailed pathophysiology remains to be further untangled [70]. Histologic and immunologic pathologies of human autopsy tissue attributed to WNV show alterations of the gray matter, including neuronal necrosis with infiltrates of microglia and polymorphonuclear leukocytes, perivascular cuffing, neuronal degeneration, neuronophagia, focal glial nodules, and astrogliosis [59,70]. In human astrocytes and neurons, WNV replicates efficiently but distinctively with a higher and faster replication rate in astrocytes [58]. Interestingly, data on the viability of WNV-infected astrocytes show somehow conflicting results, even though the same strain (WNV-NY99) was used for infection. For instance, although the demise of primary human fetal astrocytes was clearly faster than that of primary human fetal neurons, a human astrocytic (U373) cell line did not show any signs of apoptotic cell death in contrast to a neuronal (LAN-2) cell line [58,70]. The differences may arise from the use of different viral strain passages, the use of different cell models, and from sampling at different times after infection. Similar to data obtained in primary human astrocytes, WNV also caused astrocyte activation and cell death in a mouse brain slice culture [72]. On the other hand, new-born primary mouse astrocytes were prone to cell death to some extent after WNV infection; some resisted cell death despite clearly observed alterations in cell morphology or a cytopathic effect (CPE). Moreover, CPE evolved more slowly in astrocytes than in neurons [73]. Importantly, of particular interest is the finding that primary murine astrocytes in culture established persistent WNV infection, suggesting that these cells may be involved in chronic or persistent WNV infection in the CNS [73].

Astrocytes have an active role in the spread of WNV in the CNS, and in maintenance of the neuroinvasive potential of WNV. Both processes are strain dependent as elucidated by a study of highly virulent and avirulent strains, which revealed differences in the WNV dissemination profile within the CNS [74]. Replication of two different WNV strains (avirulent and neuropathogenic) had different replication dynamics in human brain cortical astrocytes, whereas in neurons, the dynamics were similar for both strains. The lower replication rate of an avirulent strain in astrocytes was not attributed to the action of IFNs, but to astrocyte-specific restriction of WNV particle production through furin-like proteases [74,75]. Thus, astrocytes represent an attractive target for cell-specific amelioration of WNV-induced neuropathology [74].

In addition to CPE and the expression of proteins for specific restriction of WNV particle production, other physiologic and morphologic changes are also triggered in astrocytes. Among them is the expression of endoplasmic reticulum stress-related genes linked to WNV neurovirulence [70]. In human WNVE cases, endoplasmic reticulum stress-related genes were markedly higher, especially in astrocytes [70]. This finding is of particular interest in view of several studies showing that different viral infections play a role in endoplasmic reticulum stress and consequently in both protective and pathogenic courses of neurologic infections [76,77]. Altogether, more than 20 genes linked to the activation of WNV-induced IFN signaling were highly upregulated, including those encoding GTPases, antiviral proteins, immune cell attractants, and proteins involved in the regulation of IFN signaling [72].

WNV-infected astrocytes also upregulate the expression of several chemokines, but only after infection with replication competent virus and not with inactivated virus [58,70,78]. Similarly, in patients with WNVE, upregulation of pro-inflammatory genes, including CCL2, CXCL10, IL-1 β, and indolamine-2’,3’-deoxygenase, was detected [70]. The role of specific chemokines in WNV infection of astrocytes is considered multifaceted; e.g., on the one hand, CXCL10 is reported to be neuroprotective; on the other hand, it shows neuropathogenic potential by triggering apoptosis [70,78].

An increase in IFN levels was detected in WNV-infected human brain cortical astrocytes, which was especially prominent in cells infected with a highly pathogenic strain of WNV [74]. In general, type I IFNs promote the expression of antiviral molecules in response to virus infections by inducing signaling cascades that culminate in the expression of proteins that block all steps of the host cell viral cycle, from entry to egress, contributing to alleviating neuropathogenesis in the CNS [79]. In agreement with this, high levels of IFN-β suppressed viral spread and replication in astrocytes [74]. IFNα/β receptor (IFNAR) signaling appears to be region specific, because WNV-infected astrocytes from distinct brain regions showed a different pattern of neuropathogenicity in mice brain. For instance, in hindbrain, enhanced BBB permeability, early viral neuroinvasion, and increased immunopathologic neuronal cell death were observed in the absence of IFNAR signaling in astrocytes [80].

Further studies are required to assess the effects of WNV on BBB permeability, especially in view of the specific chemokines and cytokines that are upregulated in WNV-infected astrocytes. In general, the production of inflammatory cytokines, including TNF-α, IL-6, IL-1β, and IFN-γ, is linked to altered BBB permeability, and it appears to be brain-region specific during virus infection, because it was reported to be more extensive in the cerebellum than in the cerebral cortex [81].

It appears that WNV triggers a kind of biphasic response in BBB permeability, tightly dependent on type I IFN responses [82]. Further research should focus on astrocytes, given that WNV infection of astrocytes specifically, and not direct infection of brain endothelial cells, was linked to altered permeability of the BBB [67] (Table 2).

### 2.3. Zika Virus

ZIKV is a mosquito-borne flavivirus that was first isolated in 1947 from a sentinel rhesus monkey in Uganda Zika forest [84]. It was first detected outside Africa and Asia in 2007 on a southwestern Pacific island, where it caused an outbreak with relatively mild syndromes, most frequently involving conjunctivitis, rash, and joint pain [84]. First extensive neurologic symptoms related to ZIKV infection were reported during ZIKV epidemics in French Polynesia, South and Central America, and the Caribbean [85,86,87,88]. Today, ZIKV has been confirmed to cause neurologic symptoms in adults (Guillain-Barré syndrome, myelitis, encephalitis, and neuralgia) and in fetuses and neonatal children (fetal growth restriction, abnormalities of the CNS, including microcephaly) caused by intrauterine infections in pregnancy [20,85,89,90,91,92]. Abnormalities of ZIKV-infected CNS become noticeable in the mid-gestation period, recognized as microcephaly, atrophy of the cerebrum, cerebellum, and brain stem, agyria, hydrocephalus, and various calcifications [20,86,92]. In addition to destruction of neuronal structures, reactive astrocytes extending into the subarachnoid space were identified in affected brain regions in brain slices from a 32-week fetus [20]. Aberrations in astrocyte functioning in the fetus may have a significant impact on the functioning of neurons during brain development. Namely, at the mid-gestation period, extensive neurogenesis and gliogenesis occurs, contributing to the growth of the fetal cerebral cortex [93,94,95,96]. Impaired functioning and growth of astrocytes at this time can contribute significantly to microcephaly. A high rate of infection of astrocytes at later developmental ages is proposed to affect damage to the cortical plate induced by prolonged infection of astrocytes, leading to spread of infection to other cortical cell types, inducing inflammation and further damage, even in uninfected cells, induced by astrocyte loss [97].

In general, ZIKV infection in neuronal cells halts cell-cycle progression, disrupts differentiation and proliferation, alters the immune response, and triggers cell death, which can all restrict fetal brain growth [26,98,99,100,101]. Any ZIKV-induced malfunctioning in astrocyte cellular processes plausibly affects brain development, because astrocytes orchestrate the functioning of neurons; e.g., they regulate synaptogenesis and integrate synaptic transmission, control the incorporation of neurons into networks, provide metabolic support for neurons, and affect brain microcirculation, the permeability of the BBB, and immune response [2,4,6,7,10,14,102,103].

The mechanisms of ZIKV-induced neuropathologies remain unclear [104]. Detection of viral antigens exclusively in microcalcifications associated with glial cells and neurons [101] makes these cell types of particular research interest. Astrocytes, together with microglia, are proposed to be major ZIKV targets in fetal brain development [26]. One of the confirmed entry routes for ZIKV infection of astrocytes is clathrin-mediated endocytosis upon binding to the Axl receptor from the Tyro3 Axl Mer (TAM) family [26]. Efficient infection of primary fetal human astrocytes was shown by several studies, starting with radial glia and, later in development, astrocytes, which proved especially vulnerable to ZIKV infection [97]. Primary fetal human astrocytes particularly stand out for their susceptibility to ZIKV infection in comparison with neurons and neural progenitor cells [97,99,104]. Different strain-dependent infection rates signify that viral genetics, where the exchange of several amino acids appears to be sufficient, affects the replication kinetics [99,104,105,106]. In addition to active viral replication, productive infection of primary fetal human astrocytes has also been confirmed [97,104,105].

ZIKV infection also triggers morphologic changes in primary human astrocytes [104,107]. African and Asian strains evoked progressive CPEs, leading to extensive cell death, including apoptosis, several days post infection [104]. Large cytoplasmic paraptosis-like vacuoles in ZIKV-infected primary human astrocytes indicate that ZIKV also triggers caspase-independent cell death [108]. In addition to vacuoles, a time-dependent increase in membranous exosomes has been reported in ZIKV-infected human fetal astrocytes [104].

As is the case for TBEV, astrocytes are also proposed to present a reservoir for ZIKV, and they apparently induce neuroinflammation through pro-inflammatory cytokines mediating synaptic and cognitive changes [107,109].

In addition to a pro-inflammatory role, astrocytes also have a role in limiting the spread of ZIKV. ZIKV infection of primary human astrocytes rapidly induced the production of type I IFN to limit viral spread and prevent virus-induced killing of the cells [32]. The expression of several pattern recognition receptors involved in the innate immune response associated with the expression of IFN-β and interferon-stimulated genes, chemokines, and activation of the inflammasome pathway were also detected [105]. Different ZIKV strains evoked diverse kinetics of the antiviral responses, i.e., African and Asian strains led to the induction of the IFN type I transcripts but differed in the timeline of the expression of innate immune response genes and upregulation of several MAP kinases and genes involved in signaling events downstream of TLRs (toll-like receptors) and RLRs (NOD-like receptors). The latter effect appears to be astrocyte specific when attributed to the Asian strain [105]. The African strain induced the primary expression of genes, whose activity has been associated with neurodegenerative disease [105].

Once ZIKV crosses the BBB and, similar to other flaviviruses, infects astrocytes, it can alter the maintenance and permeability properties of the BBB. In general, several studies have described ZIKV infection of brain endothelial cells after crossing the BBB. In *in vitro* BBB models, ZIKV infected brain endothelial cells (where the virus replicated and was released from), without compromising the integrity or permeability of the BBB [110,111]. However, in mouse embryo brain, ZIKV infection resulted in abnormal vasculature and a leaky BBB, indicating that ZIKV triggered progressive astrogliosis, which may contribute to the altered properties of the BBB [48] (Table 3).

### 2.4. Japanese Encephalitis Virus

JEV is a highly neuroinvasive flavivirus, transmitted by mosquito and widespread in Asia, Pacific Islands, and northern regions of Oceania. It has spread widely in the 20th century and in the beginning of the 21st century. Its RNA was detected, surprisingly, even in mosquitos in Italy, indicating potential introduction of JEV in Europe [34,38,112]. Although most JEV infections are asymptomatic, JEV can cause encephalitis, the most common form of viral encephalitis in the Asia-Pacific region and northern Oceania [34,113]. Approximately a third of clinical cases are fatal and around half of patients have permanent neuropsychiatric sequelae with symptoms resembling Parkinsonian syndrome, including abnormalities of movement, poliomyelitis-like paralysis, and cognitive impairment [113,114].

There are several assumptions on how JEV enters the CNS. Infection of olfactory neurons has been confirmed in macaques, suggesting one possible entry route for JEV [115]. In addition, crossing of the BBB is a confirmed pathway into the CNS. This pathway has been confirmed in mouse brain where JEV from cerebral blood vessels invaded neuronal tissue [116]. The presence of JEV was also confirmed in human brain endothelia surrounded by widespread perivascular edema [117]. JEV can be internalized into host cells via clathrin-dependent or clathrin-independent pathways, largely depending on the type of infected cells [25,118]. Details of JEV entry into astrocytes have not been defined. Once in astrocytes, JEV replicates and triggers morphologic changes in human astrocytic cell lines, although these were not evident in primary rat astrocytes [119,120]. Similar discrepancies between rat and human astrocytes, as described for TBEV infection, need to be elucidated for JEV [19,31]. Morphologic and physiologic changes in JEV-infected astrocytes may have significant consequences for infection on the CNS by JEV, consistent with the view that astrocytes are important regulators of BBB permeability. As is the case in TBEV infection, JEV also first invades the CNS, likely via astrocytes, and then affects BBB permeability by inducing viral replication and production of inflammatory cytokines and chemokines [121]. Once more, astrocytes have been shown to be an important player in altered BBB permeability; on infection with JEV, they start to release vascular endothelial growth factor (VEGF), IL-6, and matrix metalloproteinases [119,122].

In addition to affecting the BBB, astrocytes are also involved in inflammatory responses in JEV-infected CNS, because the overall levels of cytokines/chemokines in the brain increase dramatically after JEV infection [121]. Details of the contribution of astrocytes to inflammatory responses due to JEV infection are still lacking, although astrocyte production of cytokines/chemokines has been described on several occasions. An increase in different cytokines and chemokines was recorded in SVG and U87 cell lines [120]. JEV-infected human and mouse primary astrocytes release cytokines, among them IP-10, which is abundant in the early stage of infection and it mediates breakdown of the BBB together with TNF-α [123,124,125]. In addition to directly affecting the expression of tight junction proteins and consequently affecting the permeability of the BBB, chemokines released from astrocytes might play a role in the recruitment of immune cells [123,126].

Similar to TBEV and WNV, the response of astrocytes to JEV infection includes the production of type I IFN, which limits the spread of the virus and prevents virus-induced killing of the cells, as shown in mouse astrocytes [32]. This is consistent with the view that astrocytes are a major source of IFNs that protect neighboring astrocytes and neurons soon after infection [56]. Therefore, the capacity for neural repair is hampered not only if differentiated astrocytes are infected with JEV but also in the case of infection of neural stem/progenitor cells that are destined to differentiate into astrocytes or neurons [127].

In summary, JEV is an important neurotropic virus that hypothetically endangers almost half of the human population with the potential to spread throughout the world through its mosquito host. Increasing evidence is linking astrocytes to JEV neuropathology, and we are beginning to elucidate their role in enhancing and alleviating the devastating impact of JEV on neurons (Table 4).

## 3. Conclusions

Knowledge of flavivirus pathogenicity in the brain is fragmented, especially in view of the contribution of glial cells, and specifically astrocytes. Awareness of their role in spreading or lessening pathogenicity is only emerging. Because astrocytes integrate numerous homeostatic functions in the CNS, their infection inevitably affects neighboring cells, especially neurons. Recent research shows that astrocytes are important for retention and multiplication of the virus in the brain and modulation of immunoinflammatory processes. Although flaviviruses have not been linked to any devastating neurodegenerative diseases, they might play an important role in chronic neurologic dysfunction that persists after infection with several neurotropic flaviviruses.

Therapeutic approaches that will target astrocytes as a potential reservoir and important immunomodulatory cells might present an essential therapeutic opportunity for neuroprotection and development of novel antiviral therapeutic strategies.

## Figures and Tables

**Table 1:** Confirmed effects of tick-borne encephalitis virus (TBEV) infection on astrocytes and whole brain.

TBEV	Confirmed Infection	CPE	Cell Death	Effect on Blood–Brain Barrier	Chemokines/Cytokines	Traffic of Endosomes	Reference
Primary human astrocytes	+	+	+/−	+	+	N/A	[31]
Primary mouse astrocytes	+	+	+ (IFNAR knockout)	N/A	+	N/A	[32,56]
Primary rat astrocytes	+	−	−	N/A	N/A	+	[19]
Glioblastoma cell line	+	+	+	N/A	N/A	N/A	[18]
Human brain	+	+	+	N/A	N/A	N/A	[57]
Mouse, vole, shrew brain	+	N/A	N/A	+	+	N/A	[39,40,50,52]

CPE, extensive morphologic changes in the cytoplasm/cytopathogenic effect. Chemokines/cytokines, detected release of chemokines and cytokines from astrocytes. IFNAR, IFNα/β receptor; N/A, not applicable (not reported); +, the effect or upregulation was detected; −, the effect was absent; +/−, both effects observed.

**Table 2:** Reported signs of West Nile Virus (WNV) infection on astrocytes and whole brain.

WNV	Confirmed Infection	CPE	Cell Death	Effect on Blood–Brain Barrier	Chemokines/Cytokines	Traffic of Endosomes	Reference
Primary human astrocytes	+	N/A	N/A	+	+	N/A	[58,67,74,75,83]
Primary mouse astrocytes	+	+	+	N/A	N/A	N/A	[73,82]
Human astrocyte cell lines	+	N/A	−	N/A	+	N/A	[70]
Human brain	+	N/A	N/A	N/A	+	N/A	[70]
Astrocytes in mouse brain slices	+	N/A	+	N/A	N/A	N/A	[72,80]
Mouse brain	+	N/A	+	N/A	+	N/A	[78]

CPE, extensive morphologic changes in the cytoplasm/cytopathogenic effect. Chemokines/cytokines, detected release of chemokines and cytokines from astrocytes. N/A, not applicable (not reported); +, the effect or upregulation was detected; −, the effect was absent.

**Table 3:** Zika virus (ZIKV) infection induced changes in astrocytes and whole brain.

ZIKV	Confirmed Infection	CPE	Cell Death	Effect on Blood–Brain Barrier	Chemokines/Cytokines	Traffic of endosomes	Reference
Primary human astrocytes	+	+	+	N/A	+	N/A	[26,99,104,105,107,108]
Primary mouse astrocytes	+	N/A	+	N/A	N/A	N/A	[26,32]
Astrocyte cell lines	+	+	+	N/A	N/A	N/A	[97,106]
Fetal human brain, microcephaly	+	N/A	N/A	N/A	N/A	N/A	[20,101]
Fetal mouse brain, microcephaly	+	N/A	+	+	+	N/A	[48,98,99,100]
Fetal human brain cortical slices	+	N/A	N/A	N/A	N/A	N/A	[97]

CPE, extensive morphologic changes in the cytoplasm/cytopathogenic effect. Chemokines/cytokines, detected release of chemokines and cytokines from astrocytes. N/A, not applicable (not reported); +, the effect or upregulation was detected.

**Table 4:** Effects of Japanese encephalitis virus (JEV) infection on astrocytes and whole brain.

JEV	Confirmed Infection	CPE	Cell Death	Effect on the Blood–Brain Barrier	Chemokines/Cytokines	Traffic of Endosomes	Reference
Primary human astrocytes	+	N/A	N/A	+	+	N/A	[125]
Primary mouse astrocytes	+	N/A	N/A	+	+	N/A	[32,123]
Primary rat astrocytes	+	−	−	+	N/A	N/A	[122]
Astrocyte cell lines	+	+	N/A	N/A	+	N/A	[120]
Human brain	+	N/A	N/A	N/A	N/A	N/A	[117]
Mouse brain	+	N/A	N/A	+	+	N/A	[116,121]
Monkey brain	+	+	+	N/A	N/A	N/A	[115]

CPE, extensive morphologic changes in the cytoplasm/cytopathogenic effect. Chemokines/Cytokines, detected release of chemokines and cytokines from astrocytes. N/A, not applicable (not reported); +, the effect or upregulation was detected; −, the effect was absent.

## Abbreviations

BBB	Blood–brain barrier
CNS	Central nervous system
CPE	Cytopathic effect
CSF	Cerebral spinal fluid
EC	Endothelial cell
IFN	Interferon
IFNAR	IFNα/β receptor
JEV	Japanese encephalitis virus
TBEVVEGF	Tick-borne encephalitis virusVascular endothelial growth factor
WNV	West Nile virus
WNVE	WNV encephalitis
ZIKV	Zika virus
